# Protective effects of equol on the cartilage and subchondral bone in ovariectomized rats with osteoarthritis

**DOI:** 10.22038/IJBMS.2022.59036.13115

**Published:** 2022-10

**Authors:** Shao-Hua Ping, Fa-Ming Tian, Ze-Ming Zhao, Chun-Yu Liang, Fang Liu, Yu-Dan Wang, Liu Zhang

**Affiliations:** 1 Department of Orthopedic Surgery, Affiliated Hospital of North China University of Science and Technology, Tangshan, 063000, China; 2 School of Public Health, North China University of Science and Technology, Tangshan, 063000, China; 3 Department of Orthopedic Surgery, Emergency General Hospital, Beijing, 100028, China

**Keywords:** Cartilage, Equol, Osteoarthritis, Ovariectomised rats, Subchondral bone

## Abstract

**Objective(s):**

This study aimed to determine the therapeutic effect of equol (EQ) on osteoporotic osteoarthritis (OP OA).

**Materials and Methods:**

Thirty-six 12-week-old female Sprague-Dawley rats were randomly divided into sham group, OP OA group, and EQ group (n=12). OP OA was induced by anterior cruciate ligament transection (ACLT) combined with ovariectomy (OVX). EQ was orally administrated (10 μg/g/day) after the operation for 12 weeks. The efficacy was evaluated by gross pathology and histopathologic evaluation. The underlying mechanism was investigated by immunohistochemical analysis, micro-computed tomography (micro-CT) scanning, and tartrate-resistant acid phosphatase (TRAP) staining.

**Results:**

EQ effectively retarded cartilage degeneration, decreased the levels of matrix metalloproteinases-13 (MMP-13), a disintegrin and metalloproteinase with thrombospondin motifs 5 (ADAMTS-5), nuclear factor-kappa B P65 (NF-κB P65) and caspase-3, and increased the levels of collagen type II (Col-II), Col-I, aggrecan (AGG), and inhibitor of NF-κB α (IκBα) in the cartilage. In addition, EQ increased bone mineral density, improved the microstructural parameters of the subchondral bone (SB), and decreased the number of osteoclasts.

**Conclusion:**

EQ exerted a chondroprotective effect on OP OA in rats, associated with inhibition of the NF-κB signaling pathway and chondrocyte apoptosis. Furthermore, EQ showed an osteoprotective effect on SB via inhibiting osteoclastic activities.

## Introduction

Osteoarthritis (OA) is the most prevalent form of arthritis leading to loss of joint function in older adults (1). Typical pathological manifestations of OA include cartilage destruction, subchondral bone (SB) remodeling, and synovitis. The high morbidity of OA in postmenopausal women suggests a close association between estrogen deficiency and the progression of OA (2). In a clinical study, serum concentration of estradiol was found to be positively correlated to the severity of postmenopausal OA (3). In addition, estrogen deficiency accelerates cartilage degeneration and Sb remodeling in knee OA models (4-5). These results suggest that estrogen deficiency plays a promotive role in the progression of OA. Hormone replacement therapy (HRT) has been demonstrated to exert a protective effect on the cartilage in postmenopausal women (6). However, the clinical application of HRT is limited due to its complications including gynecologic tumors, stroke, and coronary disease (7, 8). 

Soy isoflavones are compounds extracted from soybeans that exert weak estrogen-like effects in osteoporotic models (9-11). Equol (EQ) is a metabolite of soy isoflavones, which is produced in the intestine of humans and mammals and has stronger estrogenic activities than other metabolites of soy isoflavone (12-15). The chondroprotective and osteoprotective effects of EQ were found in a model of rheumatoid arthritis (16), indicating the potential therapeutic role of EQ in arthritis diseases. However, the curative effect of EQ on OP OA still remains unclear. This study was designed to investigate the protective effect of EQ on the cartilage and SB in a model of OP OA. The model used in this study was induced by anterior cruciate ligament transection (ACLT) and ovariectomy (OVX), which is considered suitable for pharmacodynamic evaluation of OP OA (17-18). Our findings may be supportive of the application of EQ as an effective therapy for postmenopausal OA cases.

## Materials and Methods

***Animal handling***

A total of 36 female Sprague-Dawley rats aged 12 weeks (average body weight=264 g, Changsheng Biotechnology Co, Ltd, Liaoning, China) were housed at the medical lab of North China University of Science and Technology with the environment temperature controlled at 23 °C±1 °C and a dark-light cycle of 12 hr. Every 3 rats were housed in a 545×380×200 mm cage and given free access to water and soy-free chow (catalog number:1025, Beijing HFK Bioscience Co, Ltd, China). Animals were randomly divided into the sham group, OP OA group, and EQ group (n=12). OP OA was induced by OVX combined with ACLT. In brief, double incisions on the back were adopted to perform bilateral ovariectomies, and a medial approach was used for a right knee arthrotomy and the following ACLT in the OP OA and the EQ groups. The sham group only underwent exposure of the right ACL and bilateral ovaries. Rats in the EQ group were given EQ (Daicel Chiral Technologies Co., Ltd., Shanghai, China) by oral gavage (10 μg/g body weight/day (19), suspended in distilled water) at postoperative 72 hr for 12 weeks. Rats in the other two groups were only given isochoric distilled water. 

***Macroscopic evaluation***

Six right knees of each group were disarticulated, and the medial articular surfaces of the tibia plateau were scored as described in a previous study (20). In brief, gross lesions were graded as follows: 0=natural cartilage; 1= cartilage surface with a local discoloration or mild fibrillation; 2= cartilage erosion reaching the superficial or middle layer; 3= cartilage erosion reaching the deep layer; 4= cartilage erosion reaching the subchondral bone (SB). Each sample was graded by two observers in a blinded fashion and the average score was used for statistical analysis. The samples were then kept in 100% ethanol and sent for micro-computed tomographic (micro-CT) scanning.

***Histomorphometric evaluation***

Another six right knees of each group were fixed in 10% neutral formalin for 72 hr after harvest. Then the samples were rinsed with phosphate-buffered saline and decalcified in ethylenediaminetetraacetic acid (EDTA, 10%) for about 60 days. The decalcified samples were dehydrated in graded ethanol, transparenced in xylene, and embedded in paraffin wax. 6-μm-thick sagittal sections were serially cut from the medial tibia plateau of the knees. Three discontinuous sections were stained with Safranin O to perform an evaluation of the cartilage degeneration by two blinded investigators (21-22), according to the scoring system of Osteoarthritis Research Society International (OARSI). The average scores, which are proportional to the degree of cartilage destruction, were used for statistical analysis.

***Assessment of bone mass and the microstructure of S*** **B**

Samples were scanned by a micro-computed tomography (micro-CT) system (Skyscan1176; Bruker, Kontich, Belgium) with scanning parameters as follows: a source voltage of 80 kV, a source current of 280 μA, a rotation step of 0.50°, and a voxel size of 18 μm. The region of interest (ROI) was semi-manually drawn along the anatomical contour of the medial tibial plateau compartment (23). The volume of interest (VOI), composed of consecutive ROIs, was constructed distantly beneath the SB plate by the CT-Analyser software (Ver1.14.4.1, Skyscan, Kontich, Belgium) as described previously (23). Bone mineral density (BMD, mg/cm3) and trabecular bone volume (BV/TV, %) were measured to assess bone mass. Trabecular number (Tb.N, 1/mm), structure model index (SMI), and trabecular separation (Tb.Sp, mm) were used to evaluate SB microstructure. All parameters were calculated by using the CT Analyzer software.

***Immunohistochemical analysis***

The levels of collagen type II (Col-II), aggrecan (AGG), matrix metalloproteinases-13 (MMP-13), a disintegrin and metalloproteinase with thrombospondin motifs 5 (ADAMTS-5), caspase-3, nuclear factor-kappa B P65 (NF-κB P65) and inhibitor of NF-κB α (IκBα) in the cartilage were evaluated by immunohistochemistry, using primary antibodies as follows: Col-II (1:500) (P02458, Boster Co, Ltd, China), AGG (1:500)(GTX54920, Gene Tex, Inc., US), MMP-13 (1:500)(P23097, Boster Co, Ltd, China), ADAMTS-5 (1:500)(BA3020, Boster Co, Ltd., China), caspase-3 (1:500)( A2525, ABclonal Technology, China), NF-κB P65(1:200) (GTX102090, Gene Tex, Inc., US), and IκBα (1:200) (AF5002, Affinity Biosciences, US). In addition, the level of collagen type I (Col-I, 1:200) (BA0325, Boster Co, Ltd, China) in SB was measured to evaluate the content of the bone matrix. Immunohistochemical staining was performed following the instructions described previously (24). Briefly, the sections were deparaffinized in xylene, rehydrated in graded ethanol, and then incubated at 4 °C overnight with primary antibodies after antigen retrieval in 0.1% trypsin and suppression of endogenous peroxidase activity in 0.3% H_2_O_2_. The immunostained sections were treated according to the procedures from the HRP DAB Detection System (PV-6001 Polink-1, ZSGB-Bio Corp., China) and DAB kit (ZLI-9017, ZSGB-Bio Corp, China) before counterstaining with hematoxylin. Immunohistochemical images were captured by using an Olympus BX53 microscope (Olympus, Tokyo, Japan). The average integrated optical density (IOD/mm2) of the cartilage in the load-bearing area was calculated with the Image-Pro Plus software (version 6.0.0.260, Media Cybernetics, Inc., US) to assess the expression levels of target proteins, as described previously (24). 

***Assessment of the osteoclastic activity***

The activity of osteoclasts in SB was evaluated by tartrate-resistant acid phosphatase (TRAP) staining in accordance with the procedures provided by the supplier (Lianke Biotech, Hangzhou, China). The number of osteoclasts in SB was counted from two discontinuous sections for statistical analysis (25).

***Statistical analysis***

Data were shown as mean values ± standard deviation and statistically analyzed by using the SPSS software (Version 22.0, IBM Corp., Armonk, NY, US). The statistical significance in normal data was determined by one-way analysis of variance (ANOVA) with Fisher’s least significant difference (LSD) t-test or Dunnett’s T3 test. A non-parametric test (Kruskal-Wallis with Mann-Whitney) was performed for the scoring data analysis. *P*<0.05 was set for determination of the significant difference.

## Results

***EQ alleviated the cartilage destruction***

**Figure 1:**
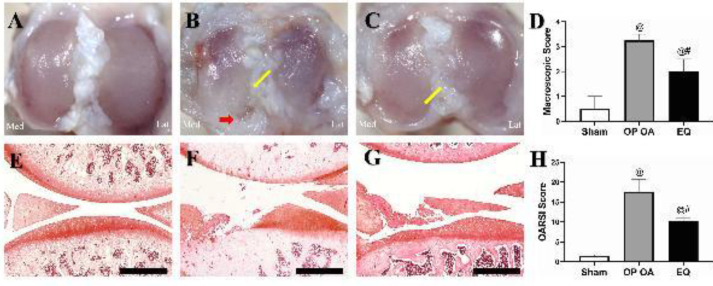
Cartilage destruction was retarded after equol (EQ) treatment. Gross evaluation: (A) Smooth articular surface of the tibia plateau in the sham group; (B) Serious cartilage erosion (yellow arrow) accompanied by exposure of the subchondral bone (red arrow) in the osteoporotic osteoarthritis (OP OA) group; (C) Cartilage erosion was alleviated after EQ treatment (yellow arrow); (D) Result of macroscopic score analysis. Safranin O staining (scale bar, 500 μm): (E) Normal cartilage of the medial tibial plateau of the sham group; (F) Cartilage defect with subchondral bone exposure in the OP OA group; (G) Alleviated cartilage erosion in the EQ group (vs OP OA group) ; (H) Result of the Osteoarthritis Research Society International (OARSI) score analysis. #*P*<0.05 vs the OP OA group; @*P*<0.05 vs the sham group

Serious degeneration in the articular cartilage of the medial tibial plateau, manifesting as discoloration, great erosion, and denudation of the cartilage (Figure 1b), was found in the OP OA group compared with the smooth cartilage surface of the sham group (Figure 1a). On the contrary, EQ effectively alleviated cartilage destruction as shown in Figure 1c. Corresponding to the gross appearance, the macroscopic score in the OP OA group was markedly increased compared with those in the EQ and sham groups (all *P*<0.05) (Figure 1d). 

***EQ decreased the histopathologic score***

The cartilage and SB in the sham group were intact (Figure 1e). However, serious cartilage defects and SB exposure were found in the OP OA group (Figure 1f). Luckily, the cartilage was efficiently preserved with a better morphology after EQ treatment (Figure 1g). Correspondingly, the OARSI score in the OP OA group was significantly higher than those in the sham and EQ groups (all *P*<0.05) (Figure 1h).

***EQ preserved BMD and ameliorated microstructural deconstruction of SB***

**Figure 2:**
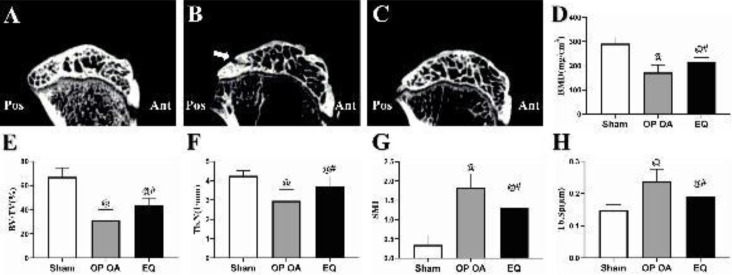
Evaluation of the subchondral bone by using micro-CT. (A) Sagittal image of the medial tibial plateau of the sham group; (B) Bone loss, loose microstructure, and a defect zone were shown in the subchondral bone (white arrow) in the osteoporotic osteoarthritis (OP OA) group; (C) Increased bone mass and improved microstructure were shown in the equol (EQ) group (vs the OP OA group); (D-H) Analysis result of the microstructural parameters including bone mineral density (BMD), bone volume fraction (BV/TV), trabecular number (Tb.N), structure model index (SMI), and trabecular spacing (Tb.Sp). #*P*<0.05 vs the OP OA group; @*P*<0.05 vs the sham group

Sagittal images of the medial tibial plateau were obtained from micro-CT scanning. The disordered and incompact microstructure and a local SB defect were shown in the OP OA group (Figure 2b) compared with the healthy SB in the sham group (Figure 2a). By contrast, EQ preserved bone mass and improved the microstructure of SB (Figure 2c). As shown in Figures 2d-h, significant decreases in BMD, BV/TV, and Tb.N, and marked increases in SMI and Tb.Sp were found in the OP OA group in comparison with those in the sham group (all *P*<0.05). These trends were significantly reversed by EQ treatment (all *P*<0.05). 

***EQ preserved the extracellular cartilage matrix (ECM) and bone collagen and inhibited*** ***overexpression of catabolic cytokines***

**Figure 3:**
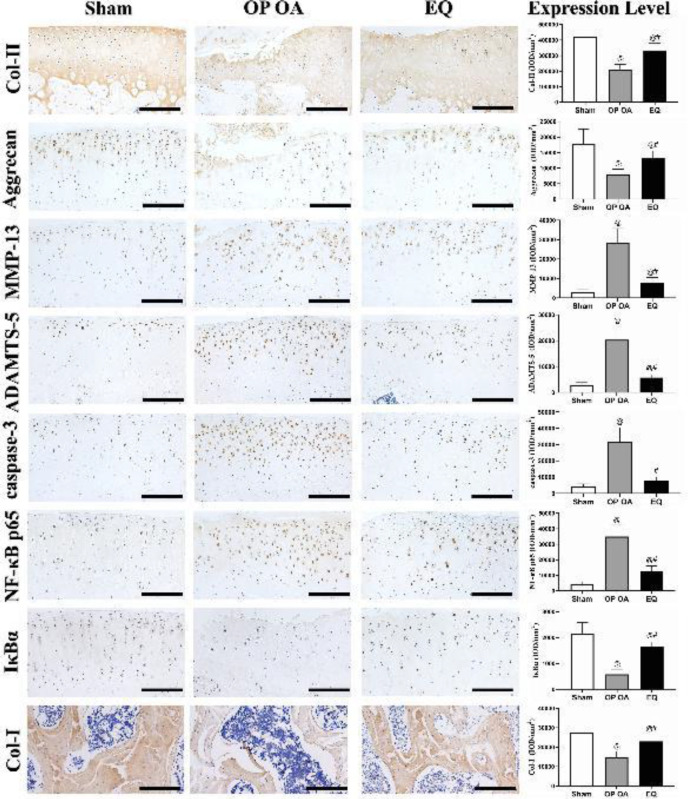
Immunohistochemical evaluation after treatment. EQ preserved the matrix of the cartilage and bone collagen and down-regulated osteoarthritis-related cytokines (scale bar, 100 μm). #P<0.05 vs the OP OA group; @P<0.05 vs the sham group. Abbreviations: ADAMTS-5: A disintegrin and metalloproteinase with thrombospondin motifs-5; Col-I: Collagen type I; Col-II: Collagen type II; IκBα: inhibitor of NF-κB α; IOD: Integrated optical density; MMP-13: Matrix metalloproteinase-13; NF-κB P65: nuclear factor-kappa B P65

The levels of MMP-13, caspase-3, ADAMTS-5, and NF-κB P65 were markedly up-regulated, and the expressions of Col-I, Col-II, AGG, and IκBα were greatly down-regulated in the OP OA group compared with the sham group (all* P*<0.05) (Figure 3). On the contrary, EQ treatment markedly reversed the expression trends of these cytokines (all *P*<0.05), suggesting that EQ preserved ECM and SB matrix, and suppressed the levels of OA-related catabolic cytokines.

***EQ reduced the number of osteoclasts in SB***

**Figure 4:**
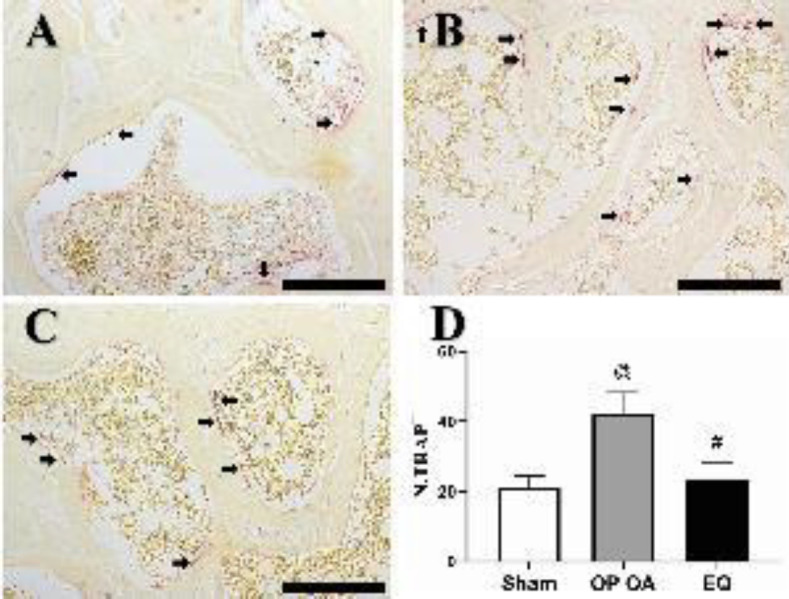
Number of osteoclasts was decreased after equol (EQ) treatment. (A) Osteoclasts (black arrow) in the sham group; (B) Number of osteoclasts was greatly increased in the osteoporotic osteoarthritis (OP OA) group; (C) EQ decreased the number of osteoclasts. (scale bar, 100 μm); (D) Analysis result of the number of TRAP+ osteoclasts (N.TRAP+). #*P*<0.05 vs the OP OA group; @*P*<0.05 vs the sham group

There is a significant increase in the number of TRAP+ osteoclasts in the OP OA group (Figure 4b) in comparison with that in the sham group (Figure 4a). In contrast, the number was significantly decreased after EQ treatment (Figure 4c) (all *P*<0.05). The relevant statistical results are shown in Figure 4d.

## Discussion

Knee OA is a progressive and incurable joint disease that is closely related to aging, hormone imbalance, and obesity (10). Estrogen deficiency increases the morbidity and intensity of OA in postmenopausal women via catabolic effects on the cartilage and SB (26). In this study, serious erosion of the articular cartilage and destruction of SB microstructure were observed in the OP OA group, as described in previous studies (4, 5). Encouragingly, EQ exerted protective effects on the cartilage and SB health. As far as we know, this is the first report on the curative effect of EQ against OP OA.

Estrogen plays an important role in maintaining the integrity of the articular cartilage, the absence of which aggravates ECM destruction and chondrocyte apoptosis, thus increasing the severity of OA (27). The main components of ECM are AGG and Col-Ⅱ, which provide critical support for the balance of cartilage metabolism (28). MMP-13 and ADAMTS-5 are the main collagenase and aggrecanase responsible for the degradation of the ECM, which are markedly overexpressed in OA cartilage (29-30). The NF-κB signaling pathway plays a crucial role in the progression of OA via up-regulating various catabolic enzymes including MMP-13 and ADAMTS-5 (28). The level of p65, the core cytokine for target gene transduction in the NF-κB signaling pathway, was increased while the level of its inhibitor IkBα was decreased in OA (31). In this study, the levels of p65, MMP-13, and ADAMTS-5 were all markedly increased while the levels of IkBα, AGG, and Col-II were decreased in the OP OA group. On the contrary, this situation was efficiently reversed after EQ treatment. These results suggested that EQ exerted a chondroprotective effect via inhibiting the expression of catabolic enzymes, which might be associated with suppression of the NF-κB signaling pathway. EQ decreased gene expression of MMP-13 and ADAMTS-5 in rheumatoid arthritis (16) and inhibited the NF-κB signaling pathway in mouse macrophages (32). These previous reports provide support for our findings. In addition, the level of caspase-3, a critical executive enzyme for chondrocyte apoptosis in OA (33), was increased in the OP OA group while it was reduced in the EQ group. This finding suggests that suppression of chondrocyte apoptosis was involved in the potential mechanism of the chondroprotective effect of EQ. 

EQ also exerted an osteoprotective effect on SB in this study. SB plays a key role in maintaining the health of the articular cartilage (34). SB and cartilage constitute an osteochondral unit to transfer loads on joints (34). However, abnormal trabecular microstructure induced by SB remodeling may result in abnormal stress focus and thereby lead to cartilage degeneration in OA (5). In this study, significant bone loss and trabecular microstructural damage were shown in the OP OA group, which is consistent with previous research (18). Bone loss may occur due to the increased osteoclastic activity after OVX according to the TRAP staining in this study, which was also reported in a model of OA induced by OVX (25). ACL injury was demonstrated to result in SB remodeling and thereby induce cartilage degeneration (35), and bone loss may reduce bone hardness and increase its deformability thus promoting the progression of trabecular microstructural destruction (34). Hence, ACLT and OVX may play synergistic roles in cartilage destruction via enhancing SB microstructural damage. Conversely, EQ inhibited osteoclastic activity, preserved bone mass, and improved SB microstructure. Inhibition of bone loss and amelioration of SB health were proved to retard the cartilage degeneration in OA (25, 34). Therefore, the osteoprotective effect of EQ, which was also demonstrated in previous studies (16, 36, 37), may be involved in the potential mechanism of its therapeutic effect on OP OA in this study. In addition, abnormal mechanic stress may trigger the activation of the NF-κB signaling pathway in the cartilage (38). Therefore, the improvement of SB microstructure, which is conducive to the stress distribution on the cartilage, may be related to the inhibitory effect of EQ on the NF-κB signaling pathway (34). However, further studies are necessary to provide direct theoretical support for this deduction.

Protective effects on cartilage and SB of EQ shown in this study indicate its therapeutic potential as an estrogen replacement in the prevention and treatment of postmenopausal OA. In previous studies (19,37,39), medication safety of EQ was confirmed. However, the role of EQ in promoting breast cancer was also reported (40,41). Therefore, EQ treatment may not be appropriate for patients in the high-risk group with breast cancer.

As a preliminary study, there are a few limitations in this study. The protein levels of OA-related cytokines were assessed by immunohistochemistry, but the corresponding gene expressions were not evaluated. In addition, the detection of nuclear translocation of p65 and phosphorylations can provide more detailed actions of the NF-κB signaling pathway. Furthermore, the pathogenesis of the OA model induced by surgery may be different from that of human OA. Therefore, further studies are necessary to determine the replicability of the therapeutic effect of EQ in clinical cases, and the potential side effects should not be neglected.

## Conclusion

EQ effectively retarded the cartilage destruction in a model of OP OA. The potential mechanism may be related to the inhibition of the NF-κB signaling pathway and caspase-3 expression, and improvement of SB health. Our findings indicate that EQ may play a potential therapeutic role in the treatment of postmenopausal OA.

## Authors’ Contributions

SHP and FMT Designed the study; SHP, ZMZ, FL, and YDW Conducted the study; SHP and ZMZ Collected and analyzed the data; SHP, FMT, CYL, and LZ Prepared the draft manuscript and critically revised the paper; SHP, FMT, ZMZ, CYL, YDW, FL, and LZ Approved the final version to be published.

## Statement of Ethics

The experimental approval for this study was obtained from the Laboratory Animal Ethical Committee of North China University of Science and Technology (NO. LX2018157).

## Conflicts of Interest

The authors have no conflicts of interest to declare.
